# DINCH Exposure Triggers Inflammatory, Oxidative, and Apoptotic Pathways in the Liver of Long-Evans Lactating Rats and Their Offspring

**DOI:** 10.3390/ijms252313017

**Published:** 2024-12-03

**Authors:** Lucía Íñigo-Catalina, Beatriz Linillos-Pradillo, Margret Schlumpf, Walter Lichtensteiger, Sergio D. Paredes, Lisa Rancan, Jesús A. F. Tresguerres

**Affiliations:** 1 Department of Physiology, School of Medicine, Complutense University of Madrid, 28040 Madrid, Spain; luinigo@ucm.es (L.Í.-C.); spared01@ucm.es (S.D.P.); 2 Department of Biochemistry and Molecular Biology, School of Medicine, Complutense University of Madrid, 28040 Madrid, Spain; beatlini@ucm.es; 3 GREEN Tox and Institute of Veterinary Pharmacology and Toxicology, University of Zurich, CH-8057 Zurich, Switzerland; margret.schlumpf@access.uzh.ch (M.S.); walter.lichtensteiger@access.uzh.ch (W.L.)

**Keywords:** DINCH, inflammation, oxidative stress, mitochondrial damage, apoptosis, liver, perinatal offspring

## Abstract

1,2-cyclohexane dicarboxylic acid diisononyl ester (DINCH) is a non-phthalate plasticizer used as a replacement of di(2-ethylhexyl) phthalate (DEHP) in daily usage items. It is not known whether continuous exposure to low doses of DINCH can lead to hepatic alterations, the liver being the organ responsible for its metabolism. The aim of this study was to evaluate the activation of inflammatory and apoptotic pathways in the liver of lactating dams after DINCH exposure, and whether these effects may be observed on postnatal day 6 (PND6) offspring. Two doses of DINCH were tested by oral administration to the following three groups of Long-Evans rats: control, DINCH-lower dose (LDINCH, 30 mg/kg b.w./day), and DINCH-high dose (HDINCH, 300 mg/kg b.w./day). Inflammatory mediators (IL-1β, TNF-α, NF-κB), mitochondrial transcriptional factors (PPARγ and PGC-1α), oxidative stress markers (SOD, CAT, GSSG/GSH), and components of the mitochondrial apoptotic pathway (PUMA, BAX, BAD, Bcl-2, Bcl-xL, Cytochrome c, APAF-1, Caspase-3, AIF) were assessed by the gene and protein expression in the liver of lactating dams and offspring. Exposure to LDINCH promoted the release of pro-inflammatory cytokines such as IL-1β and TNF-α and raised oxidative stress levels (GSSG/GSH), as well as increased Caspase-3 levels and reduced anti-apoptotic proteins (Bcl-2 and Bcl-xL), both in lactating dams and PND6 offspring. Thus, constant exposure to lower doses of DINCH can disrupt inflammatory and oxidant/antioxidant homeostasis, leading to hepatic tissue damage in lactating dams and having a perinatal effect in PND6 offspring.

## 1. Introduction

It is well documented that di(2-ethylhexyl) phthalate (DEHP), a plasticizer used in the manufacturing of everyday products, may lead to hepatic alterations by dysregulating the inflammatory response and promoting oxidative and mitochondrial damage [1,2]. Inflammation, which is a biological response of the immune system, is essential for tissue homeostasis [3]. Primary inflammatory stimuli, including interleukin-1β (IL-1β) and tumor necrosis factor-α (TNF-α), trigger important regulatory pathways such as the nuclear factor kappa-B (NF-κB) one [3]. However, overactivation of these pathways results in the disturbance of the oxidant/antioxidant balance, leading to an increase in oxidative stress, which can be considered a pathological mechanism that contributes to liver injury [4,5]. In addition, the generation of reactive oxygen species (ROS) together with outer mitochondrial membrane permeabilization (MOMP) may be the initial stage of mitochondrial apoptosis via both caspase-dependent and caspase-independent mechanisms [6].

Previous studies showed that DEHP led to mitochondrial damage, lipid peroxidation, and the downregulation of aminotransferases and lipid metabolism-related genes in rat hepatocytes [1,2]. Hepatic necrosis and fibrosis were observed in the liver of Sprague Dawley rats after exposure to DEHP, which has been related to the activation of pro-inflammatory and apoptotic pathways [1]. Furthermore, the impaired hepatic oxidant/antioxidant balance was assessed in DEHP-exposed rats, which is thought to mediate hepatocyte apoptosis via the intrinsic mitochondrial pathway [2,7].

Accumulating evidence about DEHP toxicity has led to strict European regulations for its use as a plasticizer [8,9], which in turn has increased efforts to develop viable replacement compounds to this endocrine disrupting chemical (EDC) [2,10]. An alternative to DEHP is the non-phthalate plasticizer 1,2-cyclohexane dicarboxylic acid diisononyl ester (DINCH) [9,11]. This alternative compound, like DEHP, seems to possess endocrine-disrupting properties that make it a xenoestrogen [12,13]. DINCH is replacing phthalates in food packaging, medical devices, toys and children’s products, flooring, and wall covering [8,14], which has led to a very significant increase in its global production and use. Consequently, increasing levels of DINCH metabolites are found in human urine samples [14].

After oral administration and further absorption at the gastrointestinal level, DINCH is hydrolyzed to monoisononyl cyclohexanedicarboxylate (MINCH), oxidized by the cytochrome P450 enzymes (CYPs), and finally cleaved to 1,2-cyclohexandicarboxylic acid (CHDA). Alternatively, UDP-Glucuronosyltransferases (UGTs) catalyze its conjugation to glucuronic acid [8,15]. The liver, where all the mentioned metabolic processes occur, seems to be more vulnerable to the effects of plasticizers. This is justified by its responsibility for the metabolism and detoxification of xenobiotics and specifically xenoestrogens, synthetic compounds which mimic the action of endogenous estrogens. Therefore, liver function is crucial in maintaining homeostasis under inflammatory and stress conditions [4]. In this sense, previous studies have reported hepatic alterations under exposure to DINCH [11,14]. It is particularly important to evaluate how exposure to different EDCs, which are either natural or synthetic compounds that can potently alter the metabolism, cellular signaling, and excretion of endogenous hormones by mimicking their mode of actions due to structural similarities, can affect development. This is because the perinatal period is one of the most sensitive windows for EDC exposure, as enzymes and detoxification pathways at the liver are still immature, which may lead to severe disruptions in offspring health [12,16].

Previous studies by our research group have demonstrated that EDCs bisphenol A (BPA) and bisphenol F (BPF) produce a toxic effect on the liver of pregnant and lactating rats and their offspring by increasing oxidative stress and inflammation and promoting apoptosis [17,18,19]. However, the toxicity of other disruptors, specifically DINCH, has been barely studied.

The aim of this work was to evaluate whether DINCH oral administration at two different doses to Long-Evans lactating rats during pregnancy could induce liver alterations by increasing inflammation, inducing oxidative stress, and triggering apoptosis. Moreover, it was studied whether this effect could also be observed in the offspring at post-natal day 6 (PND6).

## 2. Results

### 2.1. Effects of DINCH Exposure on Inflammatory Response in the Liver of Dams

**Figure 1:**
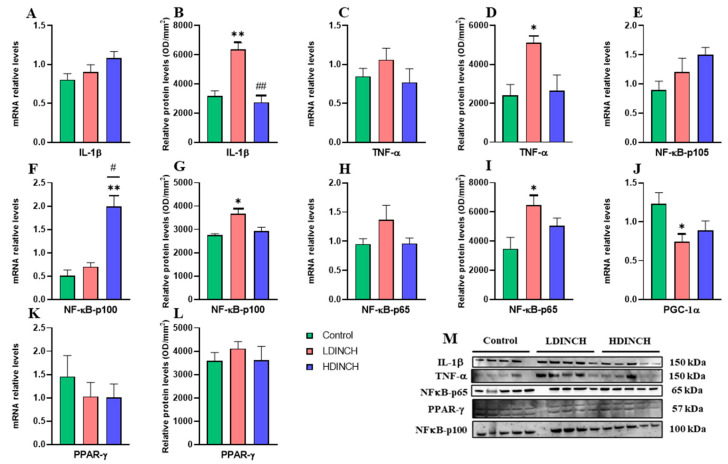
Effects of DINCH exposure on inflammatory response in the liver of dams. mRNA expression and protein levels of IL-1β (**A**,**B**), TNF-α (**C**,**D**), NF-κB-p100 (**F**,**G**), NF-κB-p65 (**H**,**I**), and PPAR-γ (**K**,**L**), and mRNA expression of NF-κB-p105 (**E**) and PGC-1α (**J**). Representative images of the Western blot results (normalized using stain-free gels) for the different proteins studied (**M**). Data represent mean ± SEM. * *p* < 0.05 vs. control, ** *p* < 0.01 vs. control. # *p* < 0.05 between treated groups, ## *p* < 0.01 between treated groups. Three groups are shown: control (in green), LDINCH (in red), and HDINCH (in blue).

No significant changes were observed in the mRNA levels of IL-1β and TNF-α due to treatment (Figure 1A,C). However, a significant increase in the protein levels of these inflammatory cytokines (Figure 1B,D) was observed in dams treated with the lower dose of DINCH. The lower dose exposure also resulted in a significant increase in protein levels of NF-κB-p100 and NF-κB-p65 subunits (Figure 1G,I). On the other hand, only NF-κB-p100 showed significantly increased gene expression in the HDINCH group (Figure 1F). In addition, differences between the treated groups were observed for IL-1β protein levels and NF-κB-p100 gene expression (Figure 1B,F).

Regarding PGC-1α, mRNA analysis (Figure 1J) revealed a significantly decreased expression of this transcriptional coactivator in dams treated with the lower dose. Further measurements of PPAR-γ, a transcriptional factor whose coactivator is PGC-1α, did not show significant results (Figure 1K,L).

Taken together, these results suggest that DINCH exposure raised some inflammatory mediators and that this increase resulted in a decreased transcriptional activity of PGC-1α in the liver of lactating dams.

### 2.2. Effects of DINCH Exposure on Antioxidant Enzyme Activities and Glutathione Concentrations in the Liver of Dams

**Figure 2:**
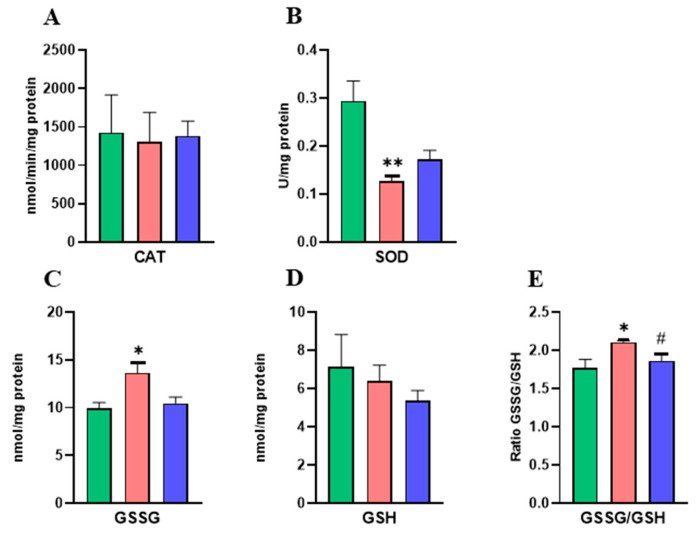
Effects of DINCH exposure on antioxidant enzyme activities and glutathione concentrations in the liver of dams. Enzymatic activity of catalase (CAT) in nmol/min/mg protein (**A**) and superoxide dismutase (SOD) in U/mg protein (**B**). Concentration of oxidized glutathione (GSSG) (**C**) and reduced glutathione (GSH) in nmol/mg protein (**D**). GSSG/GSH ratio (**E**). Data represent mean ± SEM. * *p* < 0.05 vs. control, ** *p* < 0.01 vs. control. # *p* < 0.05 between treated groups. Three groups are shown: control (in green), LDINCH (in red) and HDINCH (in blue).

It was shown that a significant decrease in SOD levels in the group of dams treated with the lower dose of DINCH compared to the control group (Figure 2B) occurred. Moreover, oxidized glutathione (GSSG) was significantly increased in the liver of LDINCH dams (Figure 2C), and so was the GSSG/GSH ratio (Figure 2E), which was also statistically significant after the comparison of both doses of DINCH.

These results suggest that DINCH exposure led to an increase in oxidative stress associated with a decrease in antioxidant activity in the liver of lactating dams.

### 2.3. Effects of DINCH Exposure on Apoptotic Markers in the Liver of Dams

**Figure 3:**
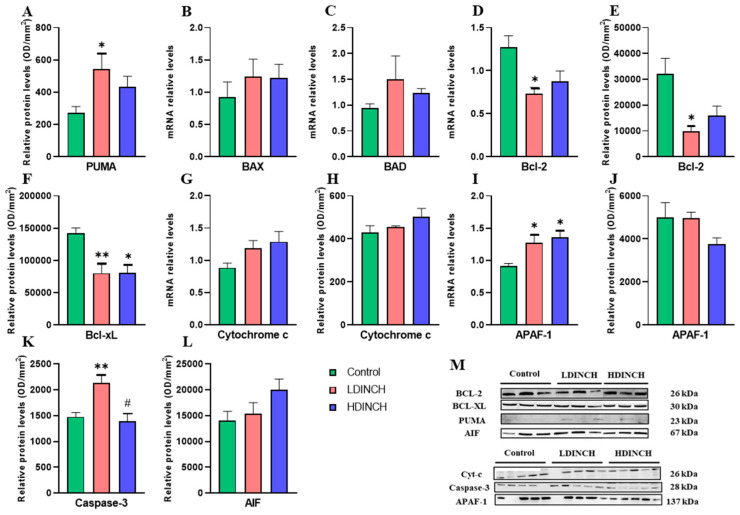
Effects of DINCH exposure on apoptotic markers in the liver of dams. Protein levels of PUMA (**A**), mRNA expression of BAX (**B**), BAD (**C**), and Bcl-2 (**D**), protein levels of Bcl-2 (**E**) and Bcl-xL (**F**), mRNA expression and protein levels of Cytochrome c (**G**,**H**), and APAF-1 (**I**,**J**), and protein levels of Caspase-3 (**K**) and AIF (**L**). Representative images of the Western blot results (normalized using stain-free gels) for the different proteins studied (**M**). Data represent mean ± SEM. * *p* < 0.05 vs. control, ** *p* < 0.01 vs. control. # *p* < 0.05 between treated groups. 3 groups are shown: control (in green), LDINCH (in red) and HDINCH (in blue).

Significantly elevated levels of the pro-apoptotic mitochondrial protein PUMA after lower dose exposure of DINCH were observed (Figure 3A), whereas no significant differences were found in BAX and BAD gene expression (Figure 3B,C). In addition, both gene and protein expression of Bcl-2 and Bcl-xL and mitochondrial anti-apoptotic and protective factors were strongly decreased, mainly in the LDINCH group (Figure 3D–F). Significantly elevated mRNA levels of APAF-1, a cytosolic Cytochrome *c* binding factor, were also observed in LDINCH dams (Figure 3I). These results are consistent with the significantly increased levels of Caspase-3 obtained after lower DINCH exposure (Figure 3K). Moreover, the levels of this apoptotic protein were statistically significant after the comparison of both doses of DINCH, with higher levels found in the liver of LDINCH-treated lactating dams (Figure 3K), implying more intense actions. Significant results for the HDINCH group were only seen for Bcl-xL (Figure 3F) and APAF-1 (Figure 3I).

Overall, these results suggest that DINCH exposure turned out to increase some of the measured pro-apoptotic mediators and strongly diminish the anti-apoptotic mediators in the liver of lactating dams. Moreover, caspase-dependent apoptosis seemed to be enhanced.

### 2.4. Effects of DINCH Exposure on Inflammatory Response in the Liver of PND6 Offspring

**Figure 4:**
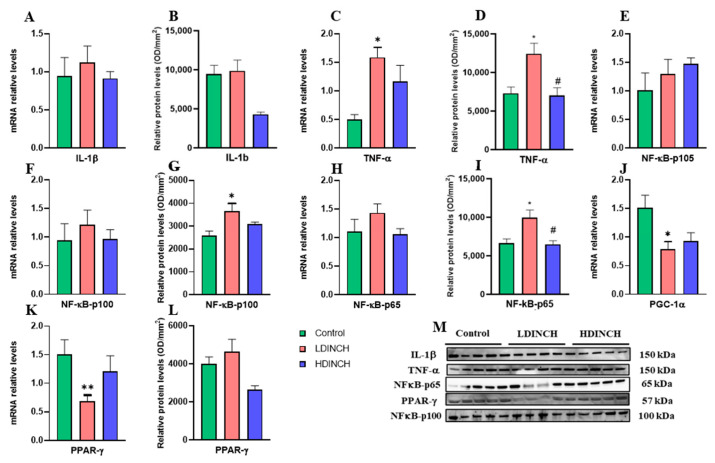
Effects of DINCH exposure on inflammatory response in the liver of PND6 offspring. mRNA expression and protein levels of IL-1β (**A**,**B**), TNF-α (**C**,**D**), NF-κB-p100 (**F**,**G**), NF-κB-p65 (**H**,**I**), and PPAR-γ (**K**,**L**), and protein levels of NF-κB-p105 (**E**) and PGC-1α (**J**). Representative images of the Western blot results (normalized using stain-free gels) for the different proteins studied (**M**). Data represent mean ± SEM. * *p* < 0.05 vs. control, ** *p* < 0.01 vs. control. # *p* < 0.05 between treated groups. Three groups are shown: control (in green), LDINCH (in red) and HDINCH (in blue).

Results showed a significant increase in the protein levels of pro-inflammatory mediators TNF-α, NF-κB-p100, and NF-κB-p65 (Figure 4D,G,I), as well as in TNF-α mRNA levels (Figure 4C). Moreover, statistically significant differences were found after the comparison of both doses of DINCH for TNF-α (Figure 4D) and NF-κB-p65 (Figure 4I), meaning more pronounced perinatal effects of lower doses of DINCH in the offspring occurred. Similarly to dams, it was observed a significantly reduced gene expression of PGC-1α in lower dose-treated offspring (Figure 4J), which was consistent with the raised NF-κB-p65 levels (Figure 4I). In addition, a significant decrease in PPAR-γ gene expression was obtained (Figure 4K).

Taken together, these results suggest that DINCH exposure augmented some inflammatory mediators, and this increase resulted in a decreased transcriptional activity of PGC-1α and PPAR-γ in the liver of PND6 offspring.

### 2.5. Effects of DINCH Exposure on Antioxidant Enzyme Activities and Glutathione Concentrations in the Liver of PND6 Offspring

**Figure 5:**
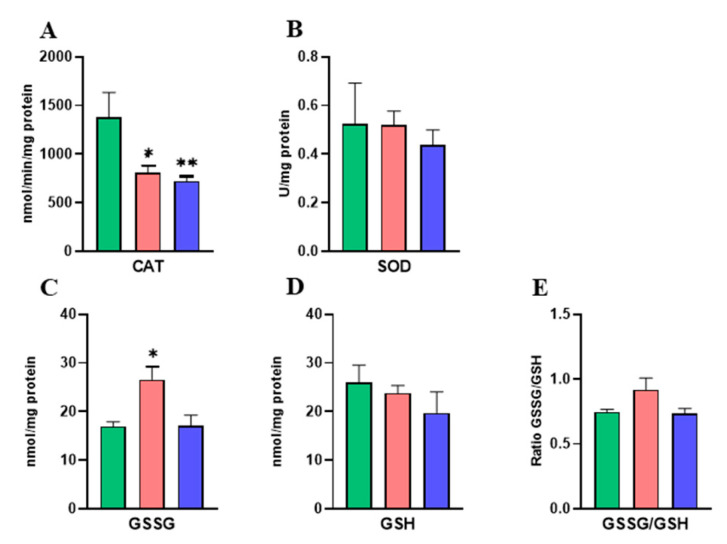
Effects of DINCH exposure on antioxidant enzyme activities and glutathione concentrations in the liver of PND6 offspring. Enzymatic activity of catalase (CAT) in nmol/min/mg protein (**A**) and superoxide dismutase (SOD) in U/mg protein (**B**). Concentration of oxidized glutathione (GSSG) (**C**) and reduced glutathione (GSH) in nmol/mg protein (**D**). GSSG/GSH ratio (**E**). Data represent mean ± SEM. * *p* < 0.05 vs. control, ** *p* < 0.01 vs. control. Three groups are shown: control (in green), LDINCH (in red) and HDINCH (in blue).

Unlike dams, pups treated with both doses of DINCH showed significantly decreased CAT activity (Figure 5A). However, no differences were observed in SOD activity levels (Figure 5B). Moreover, oxidized glutathione was significantly higher in LDINCH offspring (Figure 5C), resulting in the increasing tendency of the GSSG/GSH ratio in this group (Figure 5E).

These results suggest that DINCH exposure provoked an increase in oxidative stress associated with a decrease in antioxidant activity in the liver of PND6 offspring.

### 2.6. Effects of DINCH Exposure on Apoptotic Markers in the Liver of PND6 Offspring

**Figure 6:**
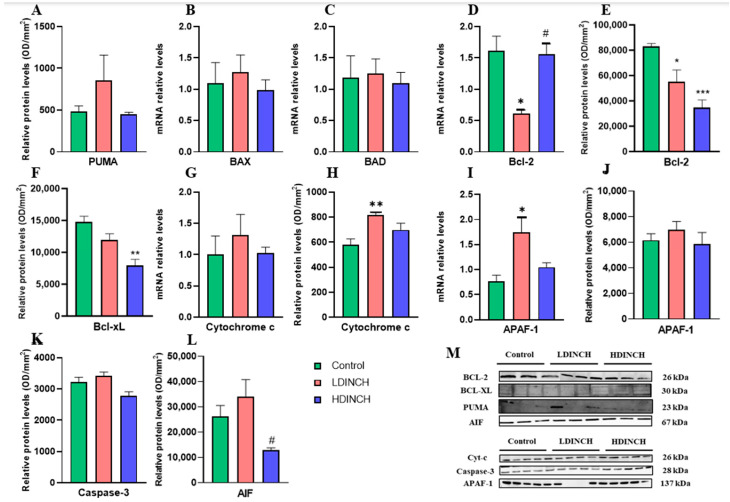
Effects of DINCH Exposure on Apoptotic Markers in the Liver of PND6 Offspring. Protein levels of PUMA (**A**), mRNA expression of BAX (**B**), BAD (**C**), and Bcl-2 (**D**), protein levels of Bcl-2 (**E**) and Bcl-xL (**F**), mRNA expression and protein levels of Cytochrome c (**G**,**H**), and APAF-1 (**I**,**J**), and protein levels of Caspase-3 (**K**) and AIF (**L**). Representative images of the Western blot results (normalized using stain-free gels) for the different proteins studied (**M**). Data represent mean ± SEM. * *p* < 0.05 vs. control, ** *p* < 0.01 vs. control, *** *p* < 0.001 vs. control. # *p* < 0.05 between treated groups. Three groups are shown: control (in green), LDINCH (in red) and HDINCH (in blue).

Mitochondrial apoptotic pathway assessment showed a very significant decrease in antiapoptotic factors Bcl-2 and Bcl-xL in pups exposed to both doses of DINCH (Figure 6D–F), being the effects on protein levels (Figure 6E,F) stronger for the high dose. These results, together with the increasing tendency of proapoptotic factors in the LDINCH group (Figure 6A–C) indicated enhanced apoptosis in liver cells in the LDINCH-exposed offspring. This was observed by significant increases in Cytochrome *c* protein levels (Figure 6H) and APAF-1 mRNA values (Figure 6I). Moreover, statistically significant differences were found in Bcl-2 and AIF mRNA levels after comparison of both doses of DINCH (Figure 6D,L), with higher levels indicating more intense actions of LDINCH in the liver of pups.

These results suggest that DINCH exposure resulted in strongly decreased anti-apoptotic mediators in the liver of PND6 offspring. Moreover, caspase-dependent apoptosis seemed to be enhanced.

### 2.7. Correlation Between Values in Dams and Pups

A moderate to strong positive correlation between the parameters analyzed in dams and pups was observed, suggesting that alterations observed in dams are often reflected in pups (Table 1). Also, strong interconnections between inflammatory, apoptotic, and oxidative stress processes for both dams and pups were observed (Table 2).

## 3. Discussion

The present study is motivated by the increasing use of 1,2-cyclohexane dicarboxylic acid diisononyl ester (DINCH) as a substitute for endocrine-disrupting phthalates in the manufacture of products whose everyday use can disturb human health due to possible contaminations. These contaminations are known to occur since DINCH metabolites have been detected in the urine samples of young adults, pregnant women, and children who, similarly to what happens with DEHP, are 5 times more exposed to DINCH than adults [12,20,21,22]. Since DINCH exposure occurs mainly through ingestion, the effects of this chemical administered orally at 2 different doses, a lower dose of 30 mg/kg (LDINCH) and a high dose of 300 mg/kg (HDINCH), were evaluated in the liver of lactating rats, and the perinatal effects on the livers of their PND6 offspring were assessed.

Prior studies of our group have revealed that low doses of other EDCs (BPA and BPF) have hepatotoxic effects on lactating Long-Evans rats and their offspring through increased inflammation, oxidative stress, and apoptosis [17,18,19]. Furthermore, exposure to phthalates has been linked to numerous adverse pregnancy outcomes, potentially through oxidative stress-mediated mechanisms; specifically, DINCH metabolites have been reported to be not only associated with increased oxidative stress but also with enhanced inflammation in pregnant women [5]. During inflammation, NF-κB reduces PGC-1α expression and activity, which leads to mitochondrial ROS accumulation [22]. Increased oxidative stress is considered a triggering mechanism of liver pathology [4]. Hence, possible alterations of pro-inflammatory pathways and hepatic oxidant/antioxidant mechanisms are the first issue addressed in this study.

In accordance with our results, DEHP-driven inflammatory processes have been observed in the liver of Sprague Dawley rats and humans [1]. Also, other authors have seen significantly upregulated inflammatory markers after DINCH exposure in the mammary gland of adult female rats [12]. Furthermore, NF-κB activation and TNF-α and IL-1β release are known to be triggered by DINCH and its metabolite MINCH in human macrophages [23].

In dams, the significant decrease observed in the expression of PGC-1α is consistent with increased NF-κB-p65 (Figure 1H,I), as low PGC-1α levels would promote NF-κB activation [21,22]. Although no alterations were seen in the measured PPAR-γ gene expression and protein levels, PPARs signaling dysregulation after DINCH exposure have also been observed by other authors in different tissues [12,24]. In addition, it has been reported that increased hepatic oxidative stress and inflammation might be reduced by PPAR-γ agonists via the regulation of NRF-2 and NF-κB pathways in rats [25,26], so that activated NF-κB (Figure 1F,G,I) would mean PPAR-γ impairment (Figure 1K,L). However, further experiments are needed to elucidate DINCH effects on PPAR-γ hepatic expression and activity, in which the translocation ratio nucleus/cytoplasm should be considered towards measuring the active fraction of this transcription factor.

Previous studies have related an inflammatory state to high levels of oxidative stress in the liver of rodents [2]. Our results exhibited alterations in hepatic oxidant/antioxidant homeostasis [4], such as SOD, which catalyzes the dismutation of superoxide anion radicals (O_2_) into hydrogen peroxide (H_2_O_2_) and molecular oxygen, was significantly decreased in dams treated with the lower dose of DINCH (Figure 2B). Moreover, an increased GSSG/GSH ratio (Figure 2E) in the LDINCH group, both with respect to the control and the HDINCH groups, may indicate that lower doses of DINCH cause oxidative damage in liver cells [4].

Similar alterations in hepatic antioxidant defense have been reported both in murine models and humans, including SOD activity depletion in the liver of Sprague Dawley rats after DEHP exposure [1] as well as modifications in SOD protein levels, especially at low doses, in rat dams and zebrafish [14,27]. Also, both DEHP and DINCH are known to induce oxidative stress on human mature adipocytes [24], and DINCH has been associated with oxidative stress, mitochondrial dysfunction, and apoptosis in human macrophages, leading to the activation of pro-inflammatory pathways [23].

Xenobiotic-induced apoptosis has been reported numerous times, and it is known that the presence of EDCs, like DINCH, may impair hepatic mitochondrial function, which, together with high ROS levels (Figure 2C,E), may result in the activation of mitochondrial apoptosis pathways [6,23]. Therefore, the levels of different components of the mitochondrial apoptotic cascade were assessed.

Although very little is known about the changes in apoptosis that take place when subjects are exposed to DINCH, previous studies have confirmed mitochondrial dysfunction and promotion of apoptosis, with underlying dysregulation of the pro-apoptotic/anti-apoptotic protein ratio [23]. Moreover, Bcl-2 downregulation (Figure 3D,E) has been linked to PPAR-γ disruptions [25,28].

In accordance with the mentioned results for dams, increased inflammation in PND6 offspring was also observed (Figure 4C,D,G,I). Likewise, we saw a decrease in hepatic antioxidant activity (Figure 5A) and a subsequent increase in oxidative stress levels (Figure 5C). Furthermore, gene expression and levels of anti-apoptotic proteins also declined in the offspring (Figure 6D–F).

These results are consistent with previous reports in which hepatic alterations and impaired liver metabolic capacity were observed in rat offspring [11]. Disturbing homeostasis by an inflammatory environment and an increased oxidant/antioxidant imbalance can potentially lead to serious or permanent effects, especially when exposure occurs during fetal development, childhood, and puberty [29].

There are not many studies in the literature showing the effects of DINCH at the offspring level, but it is very important and necessary to study exposure during the early stages of development. The prenatal period is a critical phase in which exposure to exogenous compounds can affect fetal development [29]. The fetus is extremely vulnerable due to its still immature metabolic pathways, which limit its capacity to metabolize, detoxify, and eliminate chemical compounds such as xenoestrogens [30]. Hepatic glucuronidation pathways are known to be very weak in fetal livers as well as in newborn offspring, both in rodents and humans. In addition, human neonates have lower levels of pancreatic lipase than adults, suggesting a reduced metabolic capacity in babies. Perinatal exposure and placental transfer, which in rats occurs late in gestation, can also produce developmental changes that contribute to adverse health consequences in adulthood [16,30,31,32].

In order to properly evaluate the adverse effects of EDCs and specifically DINCH, both the adult organism and offspring perinatal effects must be considered, since they may be affected in different ways due to different time windows and vulnerabilities [29,30]. Furthermore, evaluating the effects at different doses is essential. According to a study by Vanderberg et al. [33], EDCs can have effects at low doses that are not expected from the effects at higher doses. Greater intensity of effects when using lower doses is common in studies with natural hormones and EDCs. When the dose–response curves produced are non-monotonic, the effects at low doses cannot be predicted from the effects observed at higher doses. Moreover, it is also important to study the effects on both sexes under similar exposure conditions, since differences have been found for other EDCs due to variability in the metabolism, storage, and excretion of xenobiotics [34,35]. Likewise, different animal models should be employed as previous studies have shown greater vulnerability to the effects of BPF in females than males [19]. On top of all that, it is important to note that DINCH has a non-uniform composition among different production lots, which might result in varying mixtures of differentially biologically active compounds [14].

According to our results, more significant effects on inflammatory pathways, PGC-1α/NFκB-p65 interaction, antioxidant enzyme activity, and activation of mitochondrial apoptotic pathways, were observed after exposure to lower doses of DINCH in both lactating dams and offspring. In this group, the statistical association analysis revealed significant correlations between the same parameters analyzed in dams and pups, indicating that the effects of DINCH exposure observed in lactating dams are often mirrored in their offspring at postnatal day 6. In addition, strong correlations were observed between inflammatory markers such as IL1-β, TNF-α, NFκB-p65, and NFκB-p, apoptotic markers such as BCL-2, BCL-XL, CASP-3, APAF-1, and Cytc, and oxidative stress markers such as CAT, SOD, GSSG/GSH, and GSSG for both dams and pups. Overall, consistent correlations between parameters in dams and pups highlight the transgenerational effects of DINCH exposure on liver inflammation, apoptosis, and oxidative stress. However, it is important to consider that to enhance result robustness, a wider range of DINCH doses should be assessed.

The present study faces some limitations. Firstly, considering the 3Rs (Replacement, Reduction, and Refinement) principle formulated by Russel and Burch in the 1960s for more humane animal research, the study could only be performed including two doses of DINCH, since exploring a wide range of doses was not viable in terms of animal models, and it was not economically feasible either. Furthermore, the fact that the in vitro-assessed effects of EDC are not usually reproduced in vivo makes it difficult to estimate a possible dose range. Nevertheless, greater effects after exposure to lower doses could be explained by the endocrine system response to low hormone concentrations, or because response mechanisms become saturated before full receptor occupancy. Although this process is difficult to interpret, other authors have reported similar results, showing a non-monotonic dose–response relationship [36]. Our group has also observed similar non-monotonic effects with the administration of other EDCs, including BPA and BPF [17,18]. Another limitation of our study is that some of our results do not present significant differences, but they show tendences instead. This happens, for instance, when observing the results about BAD and BAX expressions in both dams and offsprings. In these cases, LDINCH animals show higher expressions than control animals, but these differences are not statistically significant. We consider that increasing the number of animals would be helpful in achieving the required statistical power.

The fact that a non-monotonic dose–response relationship exists for the effects of DINCH and the possible differences on them between sexes, together with the limited literature on the impact of this compound on health, emphasizes the need for continued research to truly elucidate the effects of low, but environmentally relevant, doses, both in adulthood and following perinatal exposure to this chemical.

Finally, since humans are constantly exposed to a large number of EDCs, and current evidence together with in vivo animal models and epidemiological studies posit a clear link between exposure to EDCs and neurodevelopmental adversity [37], the causal links between endocrine disruption and developmental neurotoxicity, which would be required for regulatory action, are still largely missing [38]. We consider that further research is needed as its results could be of interest to obstetricians, pediatricians, epidemiologists, as well as to governmental agencies.

## 4. Materials and Methods

### 4.1. Animal Model and Treatment

Eight-week-old female and ten-week-old male Long-Evans rats (Janvier Labs, Le Gen-est-Saint-Isle, France) were housed for 10 days in the animal house facilities of the School of Medicine of the Complutense University of Madrid. During this time, animals were housed in special polypropylene cages (Sodispan Research, Coslada, Madrid, Spain) that were manufactured with the lowest chemical composition of Makrolon, a polycarbonate with bisphenol A. Water bottles were made of glass. Animals were maintained at 22 ± 2 °C, with automatic light cycles (12 h light/dark), and all had free access to diet and drinking water. As previously described, this time period allowed the animals to familiarize themselves with the facilities and the staff of the animal house [17,18,19]. Animals were then randomly divided into three groups as follows: control group (non-treated), lower dose (30 mg/kg body weight/day; LDINCH) group of DINCH, and high dose (300 mg/kg body weight/day; HDINCH) group of DINCH. Doses were chosen according to preceding in vivo studies, in the context of the European project in which this study is embedded [39].

In total, 12 females and 6 males were included in the control group, 10 females and 5 males were included in the lower dose group, and 13 females and 7 males were included in the high dose group. Except for the control group, which received chow with a corresponding concentration of corn oil, all groups were fed their corresponding diet with DINCH. Food and water were fed “ad libitum”.

The animals used in this study were housed in the CAI (Animal Facility in Complutense University of Madrid) of the School of Medicine (Registration No.: ES-28079-0000086), included within the European project (H2020-SCI-BHC-2018-2020 acronym ENDpoiNTs PROEX 092/19). The project complies with the provisions of Royal Decree 53/2013 of February 1, which establishes the basic rules applicable for the protection of animals used in experimentation and other scientific purposes, including teaching.

The present investigation was approved by the Ethical Committee of Complutense University of Madrid (Madrid, Spain) and by the Autonomous Community of Madrid (Spain) (PROEX 092/19 signed on 16 October 2019) in accordance with the Guidelines for the Ethical Care of Experimental Animals of the European Union (2010/63/UE). This research is within the European project entitled “Novel Testing Strategies for Endocrine Disruptors in the Context of Developmental NeuroToxicity”, supported by the European Union’s Horizon 2020 Research and Innovation Programme (ENDpoiNTs project; grant number: 825759). All authors complied with the ARRIVE guidelines.

### 4.2. Chemicals and Experimental Design

The animal diet free of phytoestrogens was supplied by the company Granovit (Aargau, Kaiseraugust, Switzerland), and the company BASF was the supplier of 1,2-cyclohexane dicarboxylic acid diisononyl ester (DINCH) (Hexamoll). A total of 133.6 g were purchased and dissolved in ethanol and corn oil in a 10% EtOH/90% corn oil ratio. The dose ingested by each rat was calculated based on the food consumption data per animal in a pilot study, which corresponded to 7.3% of body weight.

Rats were housed in special polypropylene cages (Sodispan Research, Coslada, Madrid, Spain) and water bottles were made of glass, since it was essential to avoid the presence of bisphenols and plasticizers. A cylindrical environmental enrichment element was included, also free of EDCs. During the 2 weeks prior to mating, male and female rats were fed with a diet with the corresponding dose of DINCH. Control animals received the diet without the chemical. The mating phase occurred within each group after checking that the female was in the estrus phase. The following morning, a check for sperm-positive vaginal smear or sperm-plug was carried out and the process was repeated all mornings for 10 days. Diet treatment was maintained during pregnancy. Six females were pregnant in the control and LDINCH groups, and ten females were pregnant in the HDINCH group. After birth, the lactating dams were kept in individual cages with their offspring and dietary treatment continued until postnatal day 6 (PND6). Throughout the whole experiment (adaptation, mating, pregnancy, lactation), the cages of the control group were kept separated from the DINCH-treated groups to avoid any chance of spreading food containing DINCH that could contaminate it.

**Figure 7:**
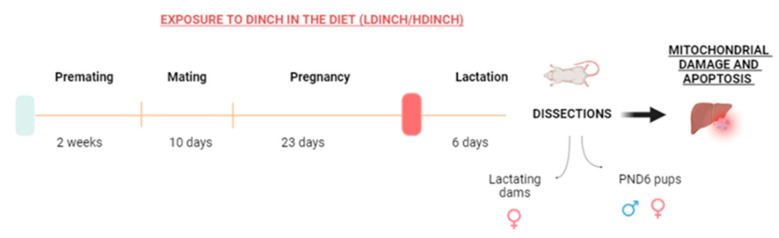
Experimental design. The diet of the parental generation was different depending on the experimental group (control, DINCH 30 mg/kg/b.w. and DINCH 300 mg/kg/b.w.). Treatment was continued until dissection. The organs were removed and preserved at −80 °C in cryotubes after applying liquid nitrogen. The sample size (N) of dams and offspring used for the experiments varied between 4 and 8 individuals per group depending on the technique used. Figure created with BioRender.com.

Lactating dams were sacrificed by decapitation using a guillotine. Female and male offspring were sacrificed at PND6 by decapitation using scissors. The livers were collected and immediately frozen in liquid nitrogen and stored at −80 °C until analysis (Figure 7).

### 4.3. RNA Isolation and Quantitative Real-Time PCR (qRT-PCR) Assessment

The total RNA was isolated from liver tissues using TRI Reagent (Sigma-Aldrich, St. Louis and Burlington, MA, USA), chloroform (Sigma-Aldrich, St. Louis and Burlington, MA, USA), isopropanol (Sigma-Aldrich, MI, USA), and cold 75% ethanol (Panreac Química, Barcelona, Spain), sequentially, with pertinent homogenization and centrifugations (13,000 rpm, 10 min, 4 °C). The RNA in the resulting pellets (frozen at −80 °C) was quantified in the Biochrom BioDrop™ UV-vis spectrophotometer (Fisher Scientific, St. Louis and Burlington, MA, USA), to determine sample concentration (μg/μL) and purity. The samples were then reverse-transcribed into cDNA using the StaRT Reverse Transcription kit from AnyGenes^®^ (AnyGenes, Paris, France). Then, qRT-PCRs were performed for *APAF-1*, *Cytochrome c*, *TNF-α*, *IL-1β*, *NF-κB*, *Bcl-2*, *BAX*, *BAD*, *PPARγ*, and *PGC-1-α* genes using a 7500 Fast Real Time PCR System thermal cycler (Applied Biosystems, Waltham, MA, USA) according to the instructions of either TaKaRa commercial company (Takara Bio Inc., Shiga, Japan) or AnyGenes commercial company (AnyGenes, Paris, France). In the case of PCRs carried out with Takara reagents (Takara Bio Inc., Shiga, Japan), the fast program was used (20″ at 95 °C followed by 40 cycles of denaturation (3″ at 95 °C) and elongation (30″ at 60 °C)), while for AnyGenes reagents, the program used was the standard (2′ at 50 °C, 10′ at 95 °C followed by 45 cycles of denaturation (10″ at 95 °C) and elongation (30″ at 60 °C). The specific primers are shown in Table 3. The amplification of the cDNA coming from the 18S ribosomal RNA was used as an endogenous control. Changes in gene expression were calculated using the 2^−∆∆CT^ [40] method. The sample size (N) varied between 6 and 8 samples from each experimental group, for both lactating dams and offspring.

### 4.4. Protein Preparation and Western Blot Analysis

Proteins were extracted from the livers with a modified RIPA lysis buffer (1× PBS, Igepal 1:100, Sodium Deoxycholate 0.5%, SDS 0.1%, PMSF 1:100, 1 mM EDTA, 1 mM EGTA), to which protease inhibitor cocktail (sigma #P-2714), PMSF (#P7626, 1 mM), sodium orthovanadate (#S6506, 2 mM), and sodium pyrophosphate (#S6422, 20 mM) were added. Samples were sonicated, quantified using the RC DC™ kit (Bio-Rad Laboratories, Richmond, CA, USA) [41], and boiled for 10 min at 100 °C in a ratio of 1:1 with gel-loading buffer (100 mmol/L Tris HCl [pH 6.8], 4% SDS, 20% glycerol, bromophenol blue 0.1, 200 mmol/L dithiothreitol). Then, 10 µL of each extract (25 µg of proteins) were subjected to SDS-PAGE using 10% Mini-PROTEAN^®^ TGX™ precast acrylamide gels (Bio-Rad Laboratories, Richmond, CA, USA). After electrophoresis, Stain-Free technology was activated using the BioRad^®^ ChemiDoc MP Imaging System (Bio-Rad Laboratories, Richmond, CA, USA) and was transferred onto a PVDF membrane using Trans-Blot^®^ Turbo™ Transfer System (Bio-Rad Laboratories, Richmond, CA, USA). Stain-Free imaging technology utilizes a polyacrylamide gel containing a proprietary trihalo compound to make proteins fluorescent directly in the gel with a short photoactivation, allowing the immediate visualization of proteins at any point during electrophoresis and Western blotting. This trihalo compound is covalently bound to tryptophan residues, enhancing their fluorescence when exposed to UV light, enabling the detection of proteins at levels as low as 10–25 ng (Bio-Rad Laboratories, Richmond, CA, USA).

After the transfer, the membranes were incubated at 37 °C for 1 h in a blocking solution composed of 5% non-fat milk in 20 mM Tris pH 7.5, 150 mM NaCl, and 0.01% Tween-20. Then, the primary antibodies for AIF, Bcl-2, Bcl-xL, PUMA, Caspase-3, Cytochrome c, APAF-1, PPAR-γ, IL-1β, NFκB-p65, NF-κB-p100, and TNF-α were incubated at 4 °C overnight. These antibodies and their dilution are shown in Table 4. Afterwards, several washing steps were performed (also with stirring) with TBS-t (10× TBS, MilliQ water and Tween-20 (Panreac Química, Barcelona, Spain)). Finally, the membranes were incubated with a polyclonal antibody conjugated to horseradish peroxidase (HRP). Once the secondary binding was completed, the membranes were washed in TBS-t with shaking.

Clarity Western ECL Substrate from Bio-Rad Laboratories (CA, USA) (#1705061) was used for development by chemiluminescence in the ChemiDoc Imaging System from Bio-Rad Laboratories (CA, USA). The bands obtained were quantified using Bio-Rad Image Lab software Image Lab 6.0. Pre-stained protein markers were used for the molecular weight determination. The measurements were normalized by the amount of protein loaded in each well (Stain-Free technology), quantifying the corresponding control.

The N of these experiments was 4 samples for each experimental group, both for dams and offspring.

### 4.5. Actioxidant Enzyme Activity

Catalase (CAT) and superoxide dismutase (SOD) activities were measured in the liver homogenates, previously lysed and sonicated with the corresponding buffers. Following Cayman Chemical kits’ instructions (Catalase Assay Kit—707002; Superoxide Dismutase Assay Kit—706002), CAT samples were homogenized in 50mM potassium phosphate buffer, pH 7.0, containing 1 mM EDTA per gram tissue; and SOD samples were homogenized in HEPES buffer, pH 7.2, containing 1 mM EGTA, 210 mM mannitol, and 70 mM sucrose per gram tissue. Then, said enzymes’ activities were analyzed spectrophotometrically according to the manufacturer’s instructions (Cayman Chemical, Ann Arbor, MI, USA), and normalized according to total liver protein content. CAT activity was determined by a chemical reaction with methanol in the presence of an optimal concentration of H_2_O_2_. The produced formaldehyde was measured spectrophotometrically using 4-amino-3-hydrazino-5-mercapto-1,2,4-triazole as chromogen at 540 nm. SOD activity was evaluated by measuring the dismutation of superoxide radicals generated by xanthine oxidase and hypoxanthine. The standard curve generated using this enzyme allows for the activity of the three types of SOD (Cu/Zn, Mn and FeSOD) to be precisely quantified.

### 4.6. Glutathione Concentrations

Liver was homogenized in phosphate buffer 50 mM and EDTA 0.1 M, pH 8. Then, 10 µL of HClO_4_ were added per mL of homogenate, and supernatants were used for the quantification of both reduced (GSH) and oxidized (GSSG) glutathione by o-phthalaldehyde (OPT) at pH 12 and pH 8, respectively, resulting in the formation of a fluorescent compound. Fluorescence was measured at 350 nm excitation and 420 nm emission [42]. Results were expressed as nmol of GSSH and GSH per mg of protein. Moreover, the GSSG/GSH ratio was calculated for each sample.

### 4.7. Statistical Analysis

Results are expressed as the mean ± SEM (Standard Error of the Mean). Due to the low sample size and the observed deviations from normality in the data distribution (assessed using Shapiro–Wilk test), a non-parametric Kruskal–Wallis test was chosen for the statistical analysis. This was followed by a Dunn-Bonferroni post hoc test for multiple comparisons to control for type I error. A reliability level of 95% was considered statistically significant (*p* < 0.05).

Moreover, in order to evaluate the associations between the various parameters measured for Long-Evans lactating rats (dams) and their postnatal day 6 (PND6) offspring (pups) exposed to DINCH, the following association statistics were performed. Data from dams and offsprings were analyzed focusing on the correlation between the same values analyzed in dams and pups, and on the association between inflammatory, apoptotic, and oxidative stress parameters for both dams and pups. Pearson correlation coefficients were calculated for each pair of variables for the dams and pups’ datasets. Then, Pearson correlation coefficients were calculated between the same variables in dams and pups to assess the consistency of DINCH exposure effects across generations. Finally, associations between inflammatory markers (IL1-b, TNF-a, NFkB-p65, NFkB-p), apoptotic markers (BCL-2, BCL-XL, CASP-3, APAF-1, Cytc), and oxidative stress markers (CAT, SOD, GSSG/GSH, GSSG) were calculated for both dams and pups. All statistical analyses were performed using Prism v8 (GraphPad Software, Inc., San Diego, CA, USA).

## 5. Conclusions

Altogether, the results of the present work indicate that DINCH oral administration at two different doses to Long-Evans rats during pregnancy and lactation can induce liver alterations in individuals exposed to DINCH, as well as in PND6 offspring after perinatal exposure. Specifically, DINCH promoted pro-inflammatory pathways, declining the activity of antioxidant liver enzymes and therefore raising oxidative stress levels. Also, this compound led to mitochondrial damage, which resulted in the activation of mitochondrial apoptotic pathways and liver damage. Although alterations were observed at both doses of DINCH, more noticeable effects occurred at the low one, resulting in an inverse dose–response relationship. Nevertheless, further research is needed to elucidate the health risk of DINCH exposure both in adult life and offspring.

## Figures and Tables

**Table 1:** Pearson correlation coefficients between dams and pups.

Marker	Correlation
IL1-β (prote)	0.65
TNF-α (prote)	0.72
NFκB-p65 (prote)	0.68
NFκB-p (prote)	0.70
PGC-1α (mRNA)	0.60
BCL-2 (prote)	0.75
BCL-XL (prote)	0.70
CASP-3 (prote)	0.78
APAF-1 (mRNA)	0.65
Cyt-c (mRNA)	0.62
CAT	0.55
SOD	0.58
GSSG/GSH	0.67
GSSG	0.64

**Table 2:** Associations between inflammatory, apoptotic, and oxidative stress parameters.

Inflammatory Markers		
	Dams	Pups
IL1-β (prot) and TNF-α (prot)	0.85	0.80
TNF-α (prot) and NFκB-p65 (prot)	0.92	0.88
NFκB-p65 (prot) and NFκB-p (prot)	0.78	0.75
**Apoptotic Markers**		
BCL-2 (prot) and BCL-XL (prot)	0.80	0.78
CASP-3 (prot) and APAF-1 (mRNA)	0.75	0.72
cytc (mRNA) and CASP-3 (prot)	0.70	0.68
**Oxidative Stress Markers**		
CAT and SOD	0.65	0.62
GSSG/GSH and GSSG	0.72	0.70
CAT and GSSG/GSH	0.60	0.58

**Table 3:** Specific primers used in the different RT-qPCRs.

Name	Primer	Sequence 5′→3′
*18S*	Forward	GGT GCA TGG CCG TTC TTA
	Reverse	TCG TTC GTT ATC GGA ATT AAC
*BAX*	Forward	GTGAGCGGCTGCTTGTCT
	Reverse	GTCCCGAAGTAGGAGAGGA
*BAD*	Forward	GCCCTAGGCTTGAGGAAGTC
	Reverse	CAAACTCTGGGATCTGGAACA
*NFκB-p65*	Forward	CGAGCTCTAAAGAGTCCCAAG
	Reverse	CCTCTGGGCCAATCAAACT
*NFκB-p100*	Forward	TGGAACAGCCCAAACAGC
	Reverse	CACCTGGCAAACCTCCAT
*NFκB-p105*	Forward	CACCTCTTCTCAAAGCAGCA
	Reverse	TCCAGGTCATAGAGAGGCTCA

Note: Missing primers (*IL-1β*, *TNF-a*, *Bcl-2*, *PPAR-γ*, *PGC-1-α*, *APAF-1*, *Cytochrome c* are the validated ones (AnyGenes^®^)).

**Table 4:** Primary antibodies used for the Western blot technique.

Antigen	Type	WB Dilution	Catalog Number	Manufacturer
AIF	RbM	1:1000	5318	Cell Signaling (Danvers, MA, USA)
IL-1β	RbP	1:7000	500-P80	PeproTech EC (London, UK)
TNF-α	RbP	1:4000	500-P72	PeproTech EC
Bcl-2	RbM	1:1000	2870	Cell Signaling
Bcl-xL	RbP	1:1000	21061	SAB (Nanjing, China)
PUMA	RbP	1:2500	GTX29643	GeneTex (Hsinchu, Taiwan)
Caspase-3	RbP	1:1000	Bs-0081R	Bioss Woburn, MA, USA
Cytochrome c	RbP	1:1000	Bs-0013R-TR	Bioss
APAF-1	RbP	1:1000	Bs-58R-TR	Bioss
PPAR-γ	RbP	1:1000	41360	SAB
NF-κB-p65	RbM	1:1000	8242	Cell signaling
NF-κB-p100	RbP	1:1000	14-6733	eBioscience (San Diego, CA, USA)

RbP: rabbit polyclonal antibody; RbM: rabbit monoclonal antibody.

## Data Availability

The data that support the findings of this study are available from the corresponding author upon reasonable request.
